# Supplementary material for: The Added Value of Intraventricular Hemorrhage on the Radiomics Analysis for the Prediction of Hematoma Expansion of Spontaneous Intracerebral Hemorrhage
Source: Diagnostics (Basel). 2022 Nov 10;12(11):2755. doi: 10.3390/diagnostics12112755 (PMC9689620; doi:10.3390/diagnostics12112755)
Supplement: Supplementary file 1 [file diagnostics-12-02755-s001.zip › Table S1_HE prediction_127 cases.pdf]

Table S1. The clinical parameters, hematoma information and short-term outcomes of 127 sICH patients based on ICH_{P+V}

	Hematoma Expansion based on ICH_{P+V}		
	Yes (58 cases)	No (69 cases)	P value
Sex			0.522
Male/Female	39/ 19 (67%/ 33%)	50/ 19 (72%/ 28%)	
Age (years)	60.8 (51, 68)	60.3 (50, 67)	0.817
Interval from onset to CT scan (min)	158 (59, 167)	207 (77, 214)	0.219
Interval between CT scans (hour)	19.3 (4.8, 24.4)	24.3 (10.1, 37.1)	0.199
Initial IPH volume (mL)	22.1 (10.1, 27.3)	17.6 (7.1, 25.0)	0.132
Initial IVH volume (mL)	2.4 (0, 2.1)	4 (0, 4.2)	0.331
Initial IPH + IVH volume (mL)	21.6 (10.6, 28.3)	24.5 (10.5, 34.1)	0.410
IPH volume change (mL)	37.8 (8.4, 63.2)	-0.3 (-1.8, 1.3)	**<0.001***
IVH volume change (mL)	5.9 (0, 9.3)	-0.2 (-0.1, 0.0)	**<0.001***
IPH + IVH volume change (mL)	43.7 (11.8, 72.0)	0 (-1.6, 1.7)	**<0.001***
IVH at baseline CT scan	23 (39.7%)	26 (37.7%)	0.820
DM	15 (25.9%)	20 (29.0%)	0.695
HTN	47 (81.0%)	56 (81.1%)	0.986
Smoking	25 (43.1%)	23 (33.3%)	0.258
Alcohol	20 (34.5%)	13 (18.8%)	**0.045***
Antiplatelet/ Anticoagulation	14 (24.1%)	9 (13.0%)	0.106
Bleeding diathesis[#]	11 (19.0%)	5 (7.2%)	**0.047***
SBP at ER > 180mmHg	31 (53.4%)	35 (50.7%)	0.760
DBP at ER> 100mmHg	35 (60.3%)	41 (59.4%)	0.916
GCS 3-12	26 (44.8%)	29 (42.0%)	0.751
Location			0.218
basal ganglia	34 (58.6%)	31 (44.9%)	
Thalamus	12 (20.7%)	19 (27.5%)	
Lobar	5 (8.6%)	13 (18.8%)	
posterior fossa	7 (12.1%)	6 (8.7%)	
Hospital stay (day)	19 (12, 29)	20 (11.8, 41.3)	**0.030***
In-hospital mortality	19 (32.8%)	4 (5.8%)	**<0.001***
Brain surgery during hospitalization	31 (53.4%)	21 (30.4%)	**0.009***
Poor outcome (mRS >3 at discharge)	55 (94.8%)	43 (62.3%)	**<0.001***

For continuous variables, median (25%, 75%) values are reported.
For number of patients, N (%) are reported.
INR >1.5, aPTT >1.5 or Platelet count <100,000/ul.
*Statistically significant difference ($p < 0.05$).
