# Supplementary figures and images for: The Added Value of Intraventricular Hemorrhage on the Radiomics Analysis for the Prediction of Hematoma Expansion of Spontaneous Intracerebral Hemorrhage
Source: Diagnostics (Basel). 2022 Nov 10;12(11):2755. doi: 10.3390/diagnostics12112755 (PMC9689620; doi:10.3390/diagnostics12112755)

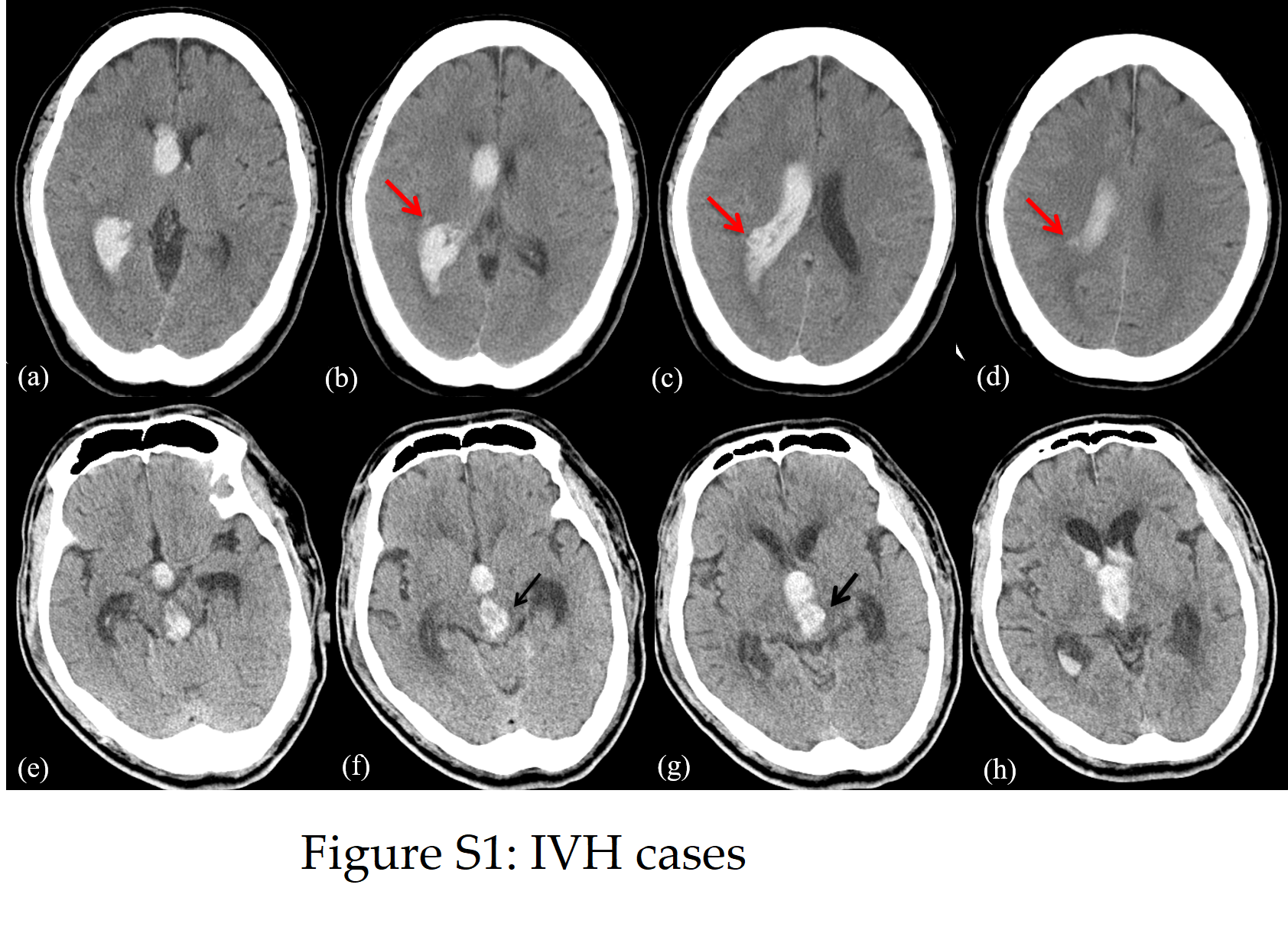

Supplement: Supplementary file 1 [file diagnostics-12-02755-s001.zip › New Fig S1_IVH cases.tif]

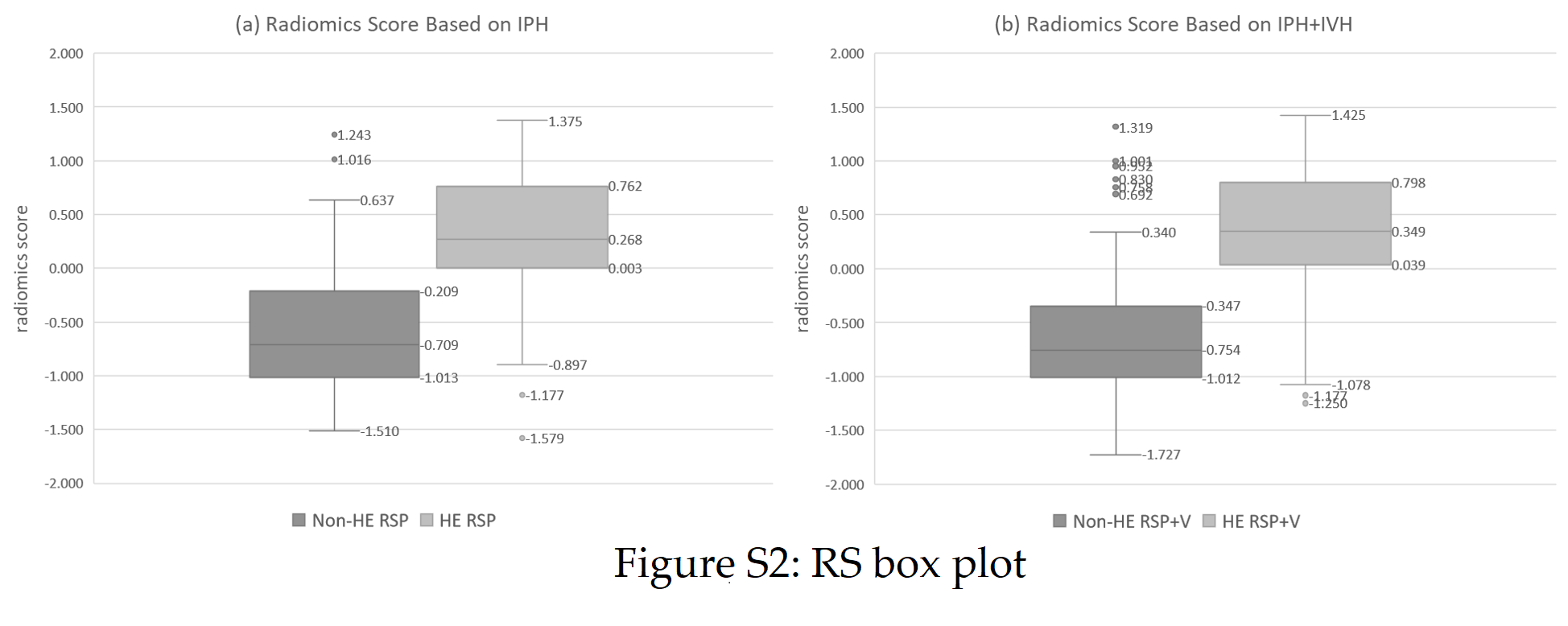

Supplement: Supplementary file 1 [file diagnostics-12-02755-s001.zip › New Fig S2_RS box plot.tif]

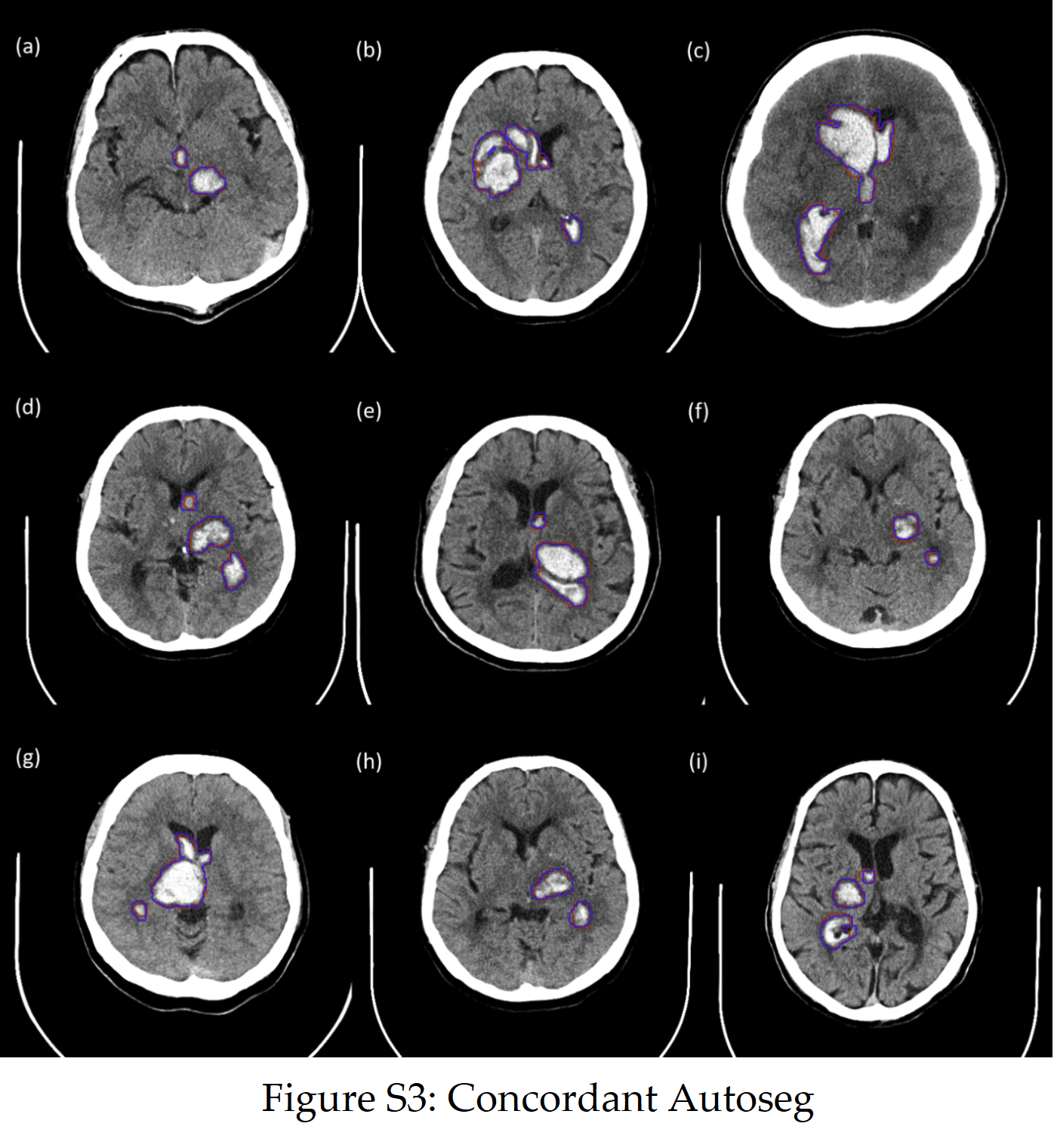

Supplement: Supplementary file 1 [file diagnostics-12-02755-s001.zip › New Fig S3_Concordant Autoseg_300dpi.tiff]

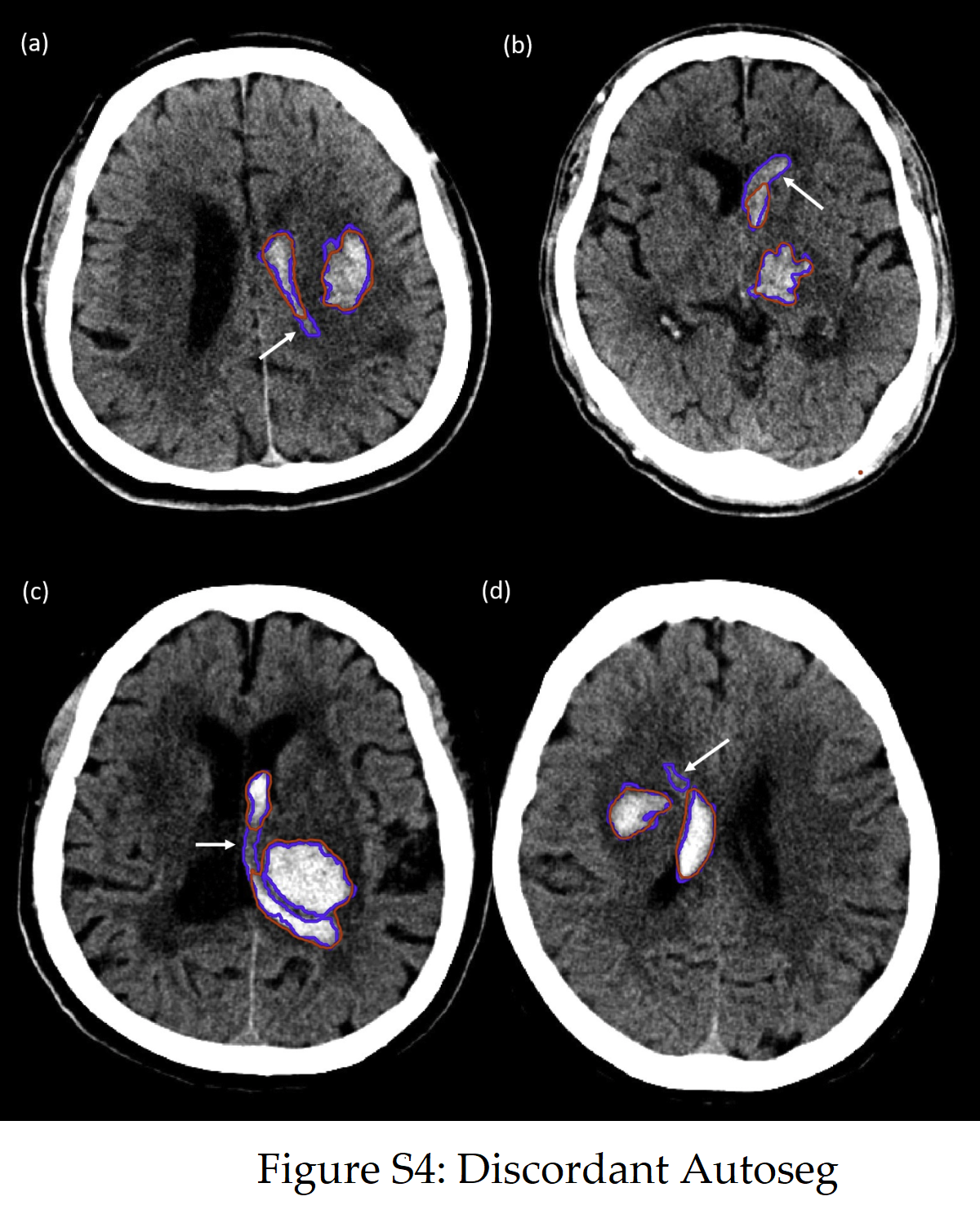

Supplement: Supplementary file 1 [file diagnostics-12-02755-s001.zip › New Fig S4_Discordant Autoseg_300dpi.tiff]
